# Noninvasive biomarkers implicated in urea and TCA cycles for metabolic liver disease

**DOI:** 10.1186/s40364-024-00694-7

**Published:** 2024-11-22

**Authors:** Guiyan Yang, Yu-Jui Yvonne Wan

**Affiliations:** 1 Department of Pathology and Laboratory Medicine, University of California, Davis, Davis Health. Room 3400B, Research Building III, 4645 2nd Ave, 95817 Sacramento, CA USA; 2 College of Veterinary Medicine, China Agricultural University, Beijing, 100193 China

**Keywords:** Liver, Metabolic disease, Machine learning, Bile acid, FXR, Gut-liver axis

## Abstract

Bile acid (BA) and its receptor FXR play crucial roles in metabolism, and dysregulated BA synthesis regulated by hepatic and bacterial enzymes causes metabolic dysfunction-associated steatohepatitis (MASH) and hepatocellular carcinoma (HCC). Moreover, because ~ 75% of hepatic blood is from the gut, liver metabolism is influenced by intestinal bacteria and their metabolites. Thus, we used gut microbiota and metabolites from the urine and serum to uncover biomarkers for metabolic distress caused by Western diet (WD) intake, aging, and FXR inactivity. Hepatic transcriptomes were profiled to define liver phenotypes. There were 654 transcriptomes commonly altered by differential diet intake, ages, and FXR functional status, representing the signatures of liver dysfunction, and 76 of them were differentially expressed in healthy human livers and HCC. Machine learning approaches classified urine and serum metabolites for differential dietary intake and age difference. Additionally, the gut microbiota could predict FXR functional status. Furthermore, FXR was essential for differentiating dietary effects in colonizing age-related gut microbes. The integrated analysis established the relationships between the metabolites and gut microbiota correlated with hepatic transcripts commonly altered by diet, age, and FXR functionality. Remarkably, the changes in metabolites involved in the urea cycle, mitochondrial metabolism, and amino acid metabolism are associated with hepatic dysfunction (i.e. FXF deactivation). Taken together, noninvasive specimens and biomarkers are promising resources for identifying metabolic distress.

**Supplementary Information:**

The online version contains supplementary material available at 10.1186/s40364-024-00694-7.

Metabolic dysfunction-associated steatotic liver disease (MASLD) is a common chronic liver disease globally. The disease spectrum ranges from simple fatty liver to severe metabolic dysfunction associated hepatitis (MASH), which may eventually progress into cirrhosis and hepatocellular carcinoma (HCC), which has a poor prognosis. It is important to identify those patients who may have severe consequences using noninvasive biomarkers [1].

Bile acids (BAs) might be the most critical metabolism stimulators jointly produced by the host and bacteria. BA receptor FXR regulates BA homeostasis via the gut-liver axis [2–6]. FXR activation suppresses inflammation and ferroptosis, whereas FXR deactivation causes dysbiosis and disrupts lipid and carbohydrate metabolism. Gut microbiota can convert primary BAs into secondary BAs, significantly shifting FXR signaling. Gut microbe-derived metabolites such as short-chain fatty acids also impact metabolism and immunity [7]. Thus, dysregulated BA synthesis or FXR inactivation results in dysbiosis, leading to the development of MASH and HCC. Additionally, human HCC consistently has reduced FXR [2–6]. We have reported that dysbiosis contributes to the progression of MASLD to HCC in a gender-specific manner in FXR knockout (KO) mice [5, 8, 9]. In addition, metabolic liver diseases, including HCC, are male-predominant, and female FXR KO mice are protected from metabolic liver disease. Therefore, FXR KO mice are human-relevant and represent excellent models for discovering the gut-liver axis biomarkers for metabolic disease prediction.

We have used urine, gut, and serum specimens to discover the biomarkers for metabolic distress caused by Western diet (WD) intake (WD vs. healthy control diet), aging (15 vs. 5 months), and FXR deactivation (FXR KO vs. wild-type mice). Hepatic transcriptomes characterized the liver phenotypes influenced by each of those metabolic risks [10, 11]. Our data showed that mice with different diets or ages had unique transcriptomes and could be clustered into distinct groups. However, when FXR was inactivated, they could no longer be grouped based on diet or age. Thus, the study establishes the essential roles of FXR in diet and age-influenced metabolic changes. Moreover, transcriptomics revealed that WD intake facilitated liver aging [10].

The study also revealed the new roles of FXR in the liver, including neuron differentiation, muscle contraction, and cytoskeleton organization. We found 654 transcripts commonly altered by differential dietary intake, age, and FXR functionality, of which 76 were differentially expressed in healthy human livers and HCC. Additional analyses for this manuscript uncovered 18 serum metabolites, 42 urine metabolites, and 26 cecal bacteria that were commonly altered due to differential dietary intake, age difference, and FXR functional status. Spearman’s correlation analysis established the relationships between those 654 hepatic transcripts and identified non-invasive biomarkers (serum and urine metabolites and gut microbiota). Figure 1 shows the correlations with coefficient values greater than 0.7. Table 1 summarizes the functions of those hepatic transcripts. Among those transcripts, only *Cyp39a*, a known HCC suppressor, and *Gm32468* were down-regulated due to FXR KO. Table 2 summarizes the sources and functions or potential roles of those metabolites or bacteria.

**Fig. 1:**
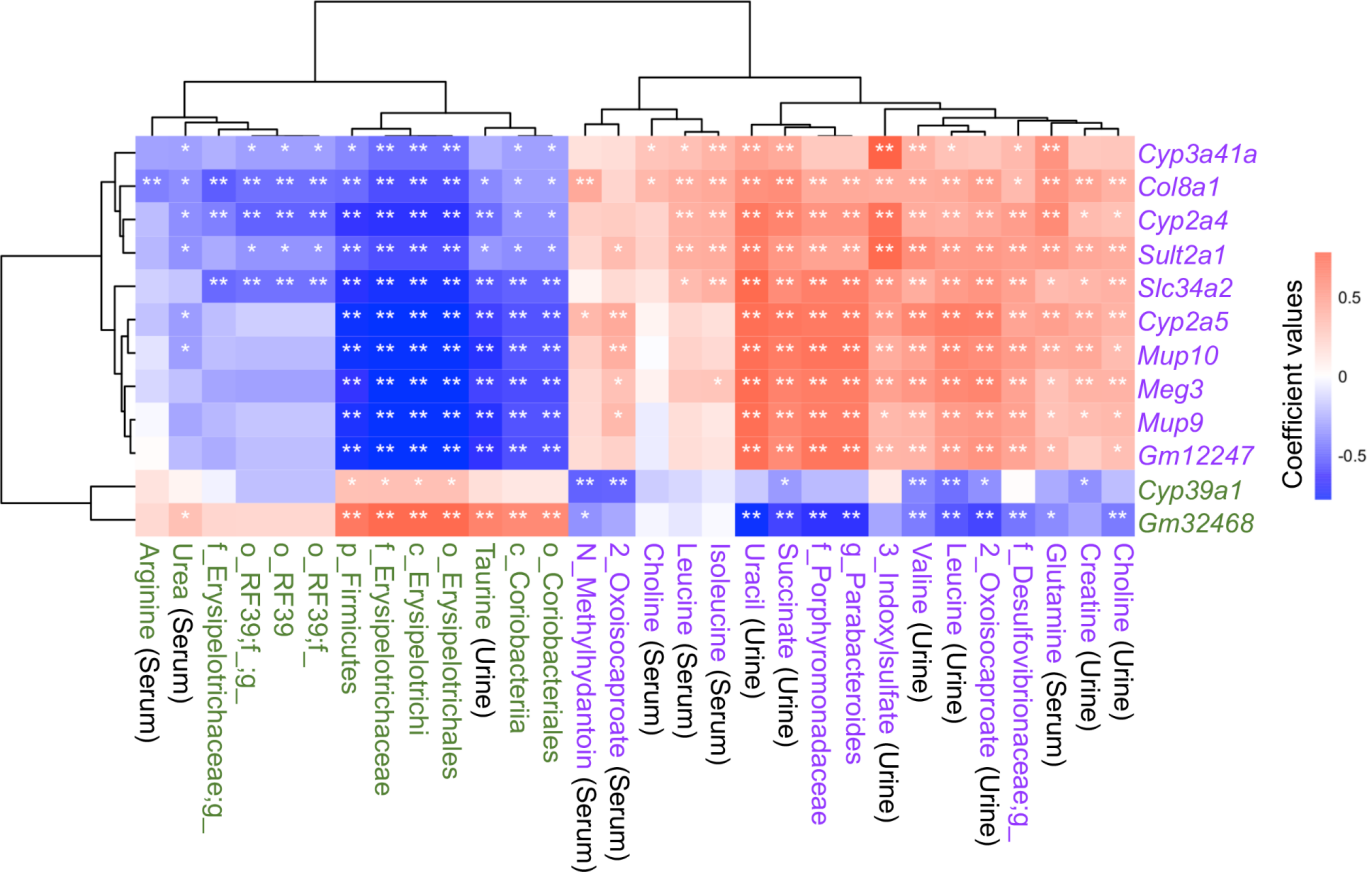
Correlation of serum and urine metabolites and gut microbiota with hepatic transcripts. Data show correlations with coefficient value > 0.7. FXR KO-upregulated features are in purple, while FXR KO-downregulated ones are in green. A significant correlation was defined when the Hochberg-adjusted *p*-value < 0.05. *p < 0.05, **p < 0.01. The bacteria at the order (o), class (c), family (f), and genus (g) levels are included.

**Table 1:** Diet, age, and FXR knockout commonly altered transcripts shown in Fig. 1

Transcript	Protein	Function	FXR KO induced change	Diseases implications
*Cyp39a1*	24-hydroxycholesterol 7-alpha-hydroxylase	Steroid metabolism. Cholesterol degradation. Lipid metabolism. Bile acid biosynthesis.	Down	CYP39A1 is an HCC suppressor in humans [16]. CYP39A1 downregulation is an HCC biomarker [17].
*Cyp3a41a*	Cytochrome P450 3A41	Catalytic activity.	Up	*Cyp3a41a* gene is downregulated in a mouse model of high-fat induced metabolic dysfunction-associated steatotic liver disease [18].
*Cyp2a4*	Cytochrome P450 2A4	Catalytic activity. Highly active in the 15-alpha-hydroxylation of testosterone. Active in the 15-alpha-hydroxylation of progesterone and androstenedione. Little or no activity on corticosterone, pregnenolone, dehydroepiandrosterone, estradiol, or estriol.	Up	*Cyp2a4* is overexpressed in chronic alcoholic liver injury in C57BL/6J mice [19].
*Cyp2a5*	Cytochrome P450 2A5	Catalytic activity. Exhibits a high coumarin 7-hydroxylase activity.	Up	CYP2A5 knockout mice are susceptible to diet-induced metabolic disorder [20].
*Sult2a1*	Sulfotransferase 2A1	Mediates the sulfation of a wide range of steroids and sterols, like pregnenolone, androsterone, DHEA, bile acids, cholesterol, and xenobiotics that contain alcohol and phenol functional groups. Catalyzes the metabolic activation of potent carcinogenic polycyclic arylmethanols.	Up	SULT2A1 is downregulated in human HCC and is correlated with poor prognosis and tumor metastasis [21].
*Slc34a2*	Sodium-dependent phosphate transport protein 2B	Involved in actively transporting phosphate into cells via Na^+^ cotransport.	Up	SLC34A2 is upregulated in HCC cell lines, and SLC34A2 knockdown inhibited HCC cell proliferation, migration/invasion, and the epithelial-mesenchymal transition phenotype [22].
*Col8a1*	Collagen alpha-1(VIII) chain	Macromolecular component of the subendothelium. Major component of the Descemet’s membrane (basement membrane) of corneal endothelial cells. A component of the endothelia of blood vessels. It is necessary for the migration and proliferation of vascular smooth muscle cells and has a potential role in maintaining vessel wall integrity and structure in atherogenesis.	Up	COL8A1 promotes the proliferation of smooth muscle cells and liver cancer cells [23].
*Meg3*	-	RNA gene (lncRNA)	Up	-
*Mup9*	-	-	Up	-
*Mup10*	-	-	Up	-
*Gm32468*	-	-	Down	-
*Gm12247*	-	-	Up	-

HCC, hepatocellular carcinoma; -, unknown

**Table 2:** Serum and urine metabolites and gut microbiota significantly correlated with those hepatic transcripts shown in Fig. 1

Metabolite (Specimen)	Source/ location	Role	FXR KO induced change
Choline (Urine, Serum)	Plants and animal organs.	A precursor of acetylcholine, a methyl donor.	Up
Creatine (Urine)	Alpha amino acids and derivatives.	Glycine, serine and threonine metabolism. Arginine and proline metabolism. Increase the expression of BDNF protein.	Up
Leucine (Urine, Serum)	Essential amino acid; meats, dairy, and soy products.	An essential branched-chain amino acid, important for hemoglobin formation. Proteinogenic α-amino acid. Valine, leucine, and isoleucine degradation.	Up
3-Indoxylsulfate (Urine)	A metabolite of the common amino acid tryptophan.	A uremic toxin. An agonist for the arylhydrocarbon receptor.	Up
Valine (Urine)	Essential amino acids; meats, dairy products, soy products.	A branched-chain essential amino acid that has stimulant activity. It promotes muscle growth and tissue repair. It is a precursor in the penicillin biosynthetic pathway.	Up
Succinate (Urine)	Exogenous food such as fruits.	Alters gene expression patterns, thereby modulating the epigenetic landscape. Exhibits hormone-like signaling functions.	Up
Uracil (Urine)	Pyrimidine in RNA. living species, ranging from bacteria to plants to humans.	An allosteric regulator and a coenzyme for many critical biochemical reactions. Pyrimidine metabolism.	Up
Taurine (Urine)	Essential amino acid; Foods such as vegetables, animal, and fish protein.	A conditionally essential nutrient, conjugates bile acids. A neurotransmitter in the brain. Taurine and hypotaurine metabolism.	Down
2-Oxoisocaproate (Urine, Serum)	An abnormal metabolite that arises from the incomplete breakdown of branched-chain amino acids.	A neurotoxin and metabotoxin.	Up
Glutamine (Serum)	Non-essential amino acid, abundantly throughout the body.	Involved in many metabolic processes. Is synthesized from glutamic acid and ammonia. The principal carrier of nitrogen in the body and is an important energy source for many cells. Proteinogenic α-amino acid. Urea cycle.	Up
Isoleucine (Serum)	Essential branched-chain aliphatic amino in many proteins.	An isomer of leucine, important in hemoglobin synthesis, blood sugar regulation, and energy levels.	Up
N-Methylhydantoin (Serum)	The product of degradation of creatinine by bacteria.	A bacterial metabolite.	Up
Urea (Serum)	Exogenous food, such as berries.	A compound formed in the liver from ammonia produced by the deamination of amino acids. It is the principal end product of protein catabolism and constitutes about one-half of the total urinary solids. Urea cycle. Arginine and proline metabolism.	Down
Arginine (Serum)	Essential L-α-amino acids.	Urea cycle. Arginine and proline metabolism.	Down
**Bacteria**	**Level**	**Role**	
Firmicutes	Phylum	Energy metabolism. Protein and amino acid metabolism. Intestinal barrier function. Immune modulation. Pathogen suppression. Vitamin synthesis. Gut motility.	Down
Erysipelotrichi	Class	Energy metabolism.	Down
Coriobacteriia	Class	Digestive tract inhabitants; SCFAs production. Immune system interactions; Anti-inflammatory effects. Competition with pathogens. Nutrient utilization.	Down
Erysipelotrichales	Order	Carbohydrate metabolism. SCFAs production. Interactions with the host immune system. Barrier function and pathogen resistance. Influence on inflammation. Competition with pathogens.	Down
Coriobacteriales	Order	Carbohydrate fermentation. SCFAs production. Immune system interactions. barrier function and pathogen resistance. Competition with pathogens. Nutrient utilization and metabolism.	Down
RF39	Order	Contribution to microbial diversity. Metabolism of substrates.	Down
Porphyromonadaceae	Family	Carbohydrate fermentation. SCFAs production. Immune system interactions. Barrier function and pathogen resistance. Competition with pathogens. Nutrient utilization and metabolism.	Up
Erysipelotrichaceae	Family	Produce broad-spectrum antibiotics. Metabolize carbohydrates.	Down
Desulfovibrionaceae	Family	Sulfate-reducing bacteria. Lipid A structures of Desulfovibrionaceae lipopolysaccharides contribute to the inflammation development.	Up
Parabacteroides	Genus	Carbohydrate metabolism and secreting SCFAs.	Up

SCFAs, short-chain fatty acids

Notably, urine choline, creatine, 2-oxoisocapronate, leucine, valine, 3-indoxylsulfate, succinate, uracil, and taurine had strong correlative coefficient values with most FXR-regulated hepatic transcripts. Among them, succinate, leucine, valine, and 2-oxoisocaproate (through their breakdown products acetyl-CoA and succinyl-CoA) are involved in the TCA cycle. Leucine, 2-oxoisocaproate, and valine contribute to ammonia production during their catabolism, and ammonia is detoxified via the urea cycle occurring in the liver.

Serum glutamate, isoleucine, leucine, and urea had high coefficient values with many FXR-regulated hepatic genes. Glutamate, isoleucine, leucine, and urea play key roles in contributing to energy production through the TCA cycle (via intermediates like α-ketoglutarate, succinyl-CoA, and acetyl-CoA), supporting nitrogen detoxification in the urea cycle, and regulating amino acid and nitrogen balance. Notably, reduced serum arginine was negatively associated with increased hepatic *Col8a1* in FXR KO mice (Fig. 1). Arginine has a substantial role in fibrosis, particularly in lung fibrosis, and both high and low levels of arginine can be associated with fibrosis [12]. Arginine is involved in nitric oxide production and collagen synthesis through its role in proline hydroxylation, an essential step in collagen formation. Thus, our data highlight the significance of the urea and TCA cycle in metabolic disease development leading to carcinogenesis. Similarly, a recent study shows that serum-based hallmarks of urea, TCA cycle, and mitochondrial derangements can predict incident fibroinflammatory liver diseases in a large group of patients nearly a decade in advance [13].

Gut bacteria such as Firmicutes (phylum), Erysipelotrichi (class), Erysipelotrichales (order), Erysipelotrichaceae (family), Coriobacteriia (class), Coriobacteriales (order), Porphyromonadaceae (family), and *Parabacteroides* (genus) had strong correlative coefficient values with most FXR-regulated hepatic transcripts. The known functions of those bacteria are summarized in Table 2.

Using machine learning approaches, we found that increased urine sucrose alone could predict WD intake with 91% accuracy, and urine metabolites (decreased creatinine and taurine and increased succinate) had 95.4% accuracy in predicting FXR deactivation [11]. Moreover, increased *Dorea*, *Dehalobacterium*, and *Oscillospira* predicted FXR deactivation with greater than 90% accuracy [11].

The biomarkers discovered in mouse models require validation in patients. A recent publication elegantly uncovered serum biomarkers IGFBP7 [Insulin-like growth factor-binding protein 7], SSc5D [Scavenger Receptor Cysteine Rich Family Member With 5 Domains], and Sema4D [Semaphorin 4D] in predicting the human MASLD fibrosis stage with high accuracy [14]. The findings were translated from a diet-induced MASLD model developed in *LDLr*^*−/−*^.*Leiden* mice.

Fecal metabolites have great potential as biomarkers for metabolic diseases since diet is the most significant dominating factor in shaping the gut microbiota. Excellent diagnostic biomarkers should be treatment targets as well. For example, high fecal deoxycholic acid (DCA) concentrations are found in obese patients and FXR KO mice. Due to the genotoxic effect of DCA, it is a cancer risk and potential diagnostic marker. Moreover, reducing bacteria-generated DCA might be a prevention or treatment option. Low concentrations of butyrate and butyrate-producing bacteria are also found in FXR KO mice. Furthermore, butyrate supplementation alleviates hepatic inflammation in FXR KO mice [8]. Thus, fecal metabolites provide helpful information to reveal individual nutritional status and can be biomarkers and treatment targets. Precision dietary supplementation based on personal gut microbiota and metabolites should be considered a future direction [15].

## Electronic supplementary material

Below is the link to the electronic supplementary material.

**Supplementary Material 1**:
**Additional file 1**.

## Data Availability

Hepatic RNA sequencing data are available on Gene Expression Omnibus (https:// www.ncbi.nlm.nih.gov/geo/) (GSE216375).

## Abbreviations

BA: Bile acid
DCA: Deoxycholic acid
FXR: Farnesoid x receptor
MASLD: Metabolic dysfunction-associated steatotic liver disease
MASH: Metabolic dysfunction associated hepatitis
HCC: Hepatocellular carcinoma
KO: Knockout
WD: Western diet
