# Supplementary material for: Noninvasive biomarkers implicated in urea and TCA cycles for metabolic liver disease
Source: Biomark Res. 2024 Nov 22;12:145. doi: 10.1186/s40364-024-00694-7 (PMC11583652; doi:10.1186/s40364-024-00694-7)
Supplement: Supplementary file 1 — Supplementary Material 1: Additional file 1. [file 40364_2024_694_MOESM1_ESM.docx]

**Supplementary Information**

**Additional file 1**

**Materials and Methods**

**Mouse Models and Data Sources**

Specific pathogen-free male wild-type (WT) and FXR KO mice were fed on either a healthy control diet (CD TD.140415; 5.2% fat, 12% sucrose, and 0.01% cholesterol, w/w, Harlan Teklad, Madison, WI, USA) or a Western diet (WD, TD.140414; 21.2% fat, 34% sucrose, and 0.2% cholesterol, w/w, Harlan Teklad, Madison, WI) since weaning and euthanized at the age of 5, 10, and 15 months. Mice were housed in 3–4 per cage in a temperature (24 °C) and light-controlled (12 h light on and off cycle) facility. Experiments were performed per the NIH Guide for the Care and Use of Laboratory Animals under protocols (#21701) approved by the Institutional Animal Care and Use Committee of the University of California, Davis. Multi-omics data were derived from these mice [1].

**Omic data analysis**

Hepatic transcriptomics (654) identified differentially expressed genes (DEGs) of due to differential diet intake, age differences, and FXR functionality as previously described [1]. From noninvasive specimens, we uncovered 18 serum metabolites，42 urine metabolites, and 26 cecal microbiota (16S rRNA sequencing, raw *p* value < 0.05) were commonly altered. DEGs were defined with FDR corrected *p*-value < 0.05 and absolute fold change ≥2. Metabolite levels were statistically significant if the uncorrected *p* value <0.05 and the false discovery rate (FDR) corrected *p* value < 0.1.

Spearman’s correlation analysis between hepatic features transcripts and non-hepatic features (serum and urine metabolites as well as gut microbiota) was performed in R 4.2.0. A significant correlation was defined when the Hochberg-adjusted *p* value <0.05. **p* < 0.05, ***p* < 0.01. Only correlation results with the coefficient value >0.7 are shown.

**References**

1. Yang GY, Jena PK, Hu Y, Sheng L, Chen SY, Slupsky CM, et al. The essential roles of FXR in diet and age influenced metabolic changes and liver disease development: a multi-omics study. Biomark Res. 2023;18;11(1):20.
